# Structural determinants underlying high-temperature adaptation of thermophilic xylanase from hot-spring microorganisms

**DOI:** 10.3389/fmicb.2023.1210420

**Published:** 2023-07-07

**Authors:** Yi Li, Hong-Qian Peng, Li-Quan Yang

**Affiliations:** ^1^College of Mathematics and Computer Science, Dali University, Dali, China; ^2^College of Agriculture and Biological Science, Dali University, Dali, China; ^3^Key Laboratory of Bioinformatics and Computational Biology, Department of Education of Yunnan Province, Dali University, Dali, China; ^4^State Key Laboratory for Conservation and Utilization of Bio-Resource in Yunnan, Yunnan University, Kunming, China

**Keywords:** xylanase, high-temperature adaptation, structural determinants, hot-spring microorganisms, protein dynamics

## Abstract

Thermophilic xylanases from hot-spring microorganisms play potential biological and industrial applications for renewable and sustainable social development. However, high-temperature adaptation mechanisms of these thermophilic xylanases remain elusive at the molecular and evolutionary levels. Here, two recently reported xylanases, named XynDRTY1 and XynM1, from hot springs were subjected to molecular dynamics (MD) simulations at a series of temperature gradients and comparatively analyzed in comparison with the evolutionary background of the xylanase family. Comparative analysis of MD trajectories revealed that the XynM1 exhibits smaller structural dynamics and greater thermal stability than the XynDRTY1, although both share a similar fold architecture with structural differences in the βα_loops. Local regions whose conformational flexibility and regular secondary structure exhibited differences as temperature increases were closely related to the high-temperature adaptation of xylanase, implying that stabilization of these regions is a feasible strategy to improve the thermal stability of xylanases. Furthermore, coevolutionary information from the xylanase family further specified the structural basis of xylanases. Thanks to these results about the sequence, structure, and dynamics of thermophilic xylanases from hot springs, a series of high-temperature-related structural determinants were resolved to promote understanding of the molecular mechanism of xylanase high-temperature adaptation and to provide direct assistance in the improvement of xylanase thermal stability.

## 1. Introduction

Xylan is the major hemicellulosic component of plant cell walls and thus becomes the second most abundant polysaccharide on earth. Xylan provides an important natural source of xylose containing functional oligosaccharides (Naidu et al., 2018). Xylans and their degradates are widely used in the production of biofuels and bio-based chemicals (Nordberg Karlsson et al., 2018). With the growing demand for renewable and sustainable social development, efficient and green production represented by enzyme catalysis has become a major trend. Xylanase (β-1,4-xylan xylanohydrolase, E.C. 3.2.1.8) which plays a crucial role in xylan depolymerization in biorefinery processes has attracted rapidly increasing attention owing to its potential biotechnological applications (Collins et al., 2005). Xylanases exhibiting high enzymatic efficiency under industrial conditions, especially at high temperatures, are extremely desired to develop effective and competitive bioprocesses for industry including paper making and animal husbandry. Therefore, the need for the discovery and characterization of thermophilic xylanases, as well as the understanding of the molecular mechanisms underlying their high-temperature adaptation, is rapidly increasing to optimize their properties and meet industrial constraints.

As natural and typical high-temperature environments, hot springs are an important source of various thermophilic novel enzymes with potential biological and industrial applications. Extreme temperature conditions force microorganisms to evolve specific molecular mechanisms, especially their internal enzymes, to maintain their biological functions in hot springs. Discovery of thermophilic xylanases from hot-spring microorganisms as an effective way to find naturally thermal mutations. Recently, a new xylanase gene, named XynDRTY1, was identified from TengChong hot springs by Yin et al. (2022). XynDRTY1 exhibits activity with natural glycosides such as beechwood with an optimal temperature of 65°C (about 338 K). Joshi et al. (2020) screened a new xylanase, called XynM1, from an aquatic habitat at extreme temperatures. Biochemical characterization revealed that the optimal temperature of XynM1 is 80°C (about 353 K), establishing a considerable thermal tolerance at high temperatures. Although a large number of novel xylanase sequences have been obtained with the development of biotechnology such as metagenomic sequencing techniques, case reports of thermophilic xylanases isolated from hot-spring microorganisms are difficult to summarize their evolutionary knowledge at the enzyme family and molecular levels. Moreover, traditional biochemical characterization in the above studies was difficult to resolve the molecular mechanism of high-temperature adaptation of thermophilic xylanases. Although efforts have been made to identify and characterize thermophilic xylanase genes from extreme environments in nature, the structural determinants underlying the thermal adaptation of thermophilic xylanases from hot-spring microorganisms remain to be further explored.

With the resolution of xylanase structures and the development of computational biology, it has become possible to probe the dynamic, kinetic, and thermodynamic properties of thermophilic xylanases at high temperatures by employing molecular dynamics (MD) simulation, which is highly useful for investigating protein structural dynamics. Through all-atom MD simulations, the molecular dynamics of biomolecules (e.g., enzymes) can be obtained to ultimately explain the “structure-dynamics-function” relationships at the atomic level (Du et al., 2016). For example, we have resolved molecular differences in the extreme conformations of the HIV envelope protein gp120 (Li et al., 2020a) and elucidated the role of ligand binding in hindering its conformational transition (Li et al., 2020b) by using MD simulations. Moreover, coevolutionary information (Morcos et al., 2014) derived from homologous families of xylanases can provide the structural constraints of thermophilic xylanases to facilitate the understanding of molecular interactions between structural features and catalytic properties.

In this study, two recently reported thermophilic xylanases, named the XynDRTY1 and XynM1, from hot springs were subjected to a series of MD simulations at increased high-temperature gradients and comparatively analyzed in the coevolutionary context of their homologous families to decipher the potential structural and dynamics determinants underlying their high-temperature adaptation. Our analysis revealed significant differences between the XynDRTY1 and XynM1 from the structural architecture, molecular dynamics, conformational flexibility, thermodynamic distribution, secondary structure evolution, and residue contact survival, explaining the different thermostabilities of two structurally similar xylanases. More importantly, residue contacts associated with the structural basis of xylanases family were identified in the xylanase family that have low sequence identity and high structural similarity, suggesting that maintaining the structural architecture will be a feasible strategy for the engineering modification of the thermophilic xylanases. Thanks to these results about the sequence, structure, and dynamics of thermophilic xylanases from hot springs, a series of high-temperature-related structural determinants were resolved to promote understanding of the molecular mechanism of xylanase high-temperature adaptation and to provide direct assistance in the improvement of xylanase thermal stability.

## 2. Materials and methods

### 2.1. Construction and comparison of structural models

The amino acid sequences of the XynDRTY1 and XynM1 were obtained from Supplementary Figure S2 in Yin et al. (2022) and Figure 2 in Joshi et al. (2020), respectively. To construct comparable structural models to the current experimental structures of xylanases, partial sequence segments of the N/C-termini were removed based on the alignment of the xylanase structures (PDB ID: 3NIY, IVBR, and IVBU, Supplementary Figures S1, S2) which were also used in the corresponding original studies. This leads to the final primary target sequences of the XynDRTY1 and XynM1 consisting of 302 and 318 residues, respectively. Due to the lack of homologous structure with high sequence identity, the AlphaFold2 (Morcos et al., 2014) implemented in the Google ColabFold notebook (Mirdita et al., 2022) was employed to construct atomic structural models. This approach builds protein models without structural templates by learning coevolutionary constraints from multiple sequence comparisons using mmseq2 (Mirdita et al., 2019). Except for the N/C terminus, the predicted Local Distance Difference Test (pLDDT) values were higher than 90% for each residue position (Supplementary Figures S3, S4) indicating that the constructed structures have high confidence. In addition, the lower Predicted Aligned Error (PAE, Supplementary Figures S5, S6) revealed that the global folding of constructed structures exhibited good stereochemical quality. The structural models of the XynDRTY1 and XynM1 were used to obtain the structure-based sequence alignment by using the Dali server (Holm, 2022). The alignment results were visualized with ESPript 3.0 (Robert and Gouet, 2014).

### 2.2. Molecular dynamics simulation

The constructed structural models of the XynDRTY1 and XynM1 were separately subjected to all-atom, μs-scale, and multiple-replica MD simulations using the GROMACS 2023 software package (Abraham et al., 2015) with the AMBER99SB-ILDN force field (Aliev et al., 2014). Each model was individually solvated using the TIP3P water molecular (Price and Brooks, 2004) in a dodecahedron box with a minimum solute-box wall distance of 1 nm and a periodic boundary condition. Counter ions were added to neutralize the net charge while obtaining a 150 mM salt concentration. After an initial steepest descent energy minimization, a series of 100-ps simulations were carried out to restrain heavy atoms by decreasing harmonic potential force constants of 1,000, 100, 10, and 0 kJ/mol/nm^2^ to effectively soak the protein into the solvent. Before the MD runs, a 400-ps pre-equilibrations simulation was performed in the NPT (isothermal-isobaric systems) ensemble without any restraint. The enzyme-solvent system was gradually heated to the targeted temperature under the constant volume over a period of 100 ps and then equilibrated under a constant pressure (1 bar) over 100 ps. To improve the conformational sampling, three independent replicas (R1-3) with 100-ns production MD simulations for each xylanase model were carried out at 300 K (26.85°C), 350 K (76.85°C), and 400 K (126.85°C) by the following protocol: atomic velocities initialized by the Maxwell distribution at corresponding temperature for each replica; leapfrog integration time was set as 2 fs due to all bonds involving hydrogen atoms were constrained using the LINear Constraint Solver (LINCS) algorithm (Hess et al., 1997); the Partial-Mesh Ewald (PME) method (Darden et al., 1993) was applied for long-range electrostatic interactions; the van der Waals and short-range electrostatics were cut off at 12.0 Å with a switch at 10.0 Å; the modified Berendsen (V-rescale) thermostat (Bussi et al., 2007) and the Parrinello-Rahman barostat (Parrinello and Rahman, 1981) were used to control the simulation temperature and pressure, respectively; the system coordinates were saved as a snapshot every 10 ps.

### 2.3. Trajectory analysis

The root means square deviation (RMSD) values of the backbone (C, C_α_, and N atoms) and the fraction of native contacts (Q) relative were calculated by using the MDTraj package (McGibbon et al., 2015) The distribution of RMSD values was calculated by histogram statistics and fitted by the Kernel Density Estimation (KDE). C_α_ root mean square fluctuations (RMSF) and secondary structure content (SSC) were extracted by “gmx rmsf” and “gmx dssp” in the GROMACS, respectively. The RMSD, Q, RMSF, and SSC were calculated to the corresponding initial structure. For each thermophilic xylanase, all MD trajectories were concatenated and were used to build a thermal distribution following a probability density function $F\left(s\right)=\ln\left(N_{i}/N_{\max}\right)$ , where $N_{i}$ is the population of bin i and $N_{\max}$ is the population of the most populated bin. The dynamical contact maps measured by the pairwise distance of C_α_ atoms were analyzed by the ConAn package.^1^

### 2.4. Coevolutionary analysis

Experimentally resolved structures of xylanase (PDB IDs listed in Supplementary Table S1) were extracted from the InterPro database^2^ with access entry IPR044846 (Glycoside Hydrolase family 10, GH-10) in March 2023. There are 202 structures in the GH-10. Only xylanase structures with amino acid sequence lengths of 300–350 residues and < 10 Å structural similarity to the XynDRTY1 or XynM1 were considered, resulting in 105 structures selected. Pairwise sequence identity and structural similarity of these structures were calculated by BioPython (Cock et al., 2009) and Prody (Bakan et al., 2011), respectively. Coevolutionary constraints were calculated using the Transformer protein language models, ESM-MSA-1b, from https://github.com/facebookresearch/esm. The script named contact_prediction.ipynb was used to predict contact probability.

## 3. Results and discussion

### 3.1. Structural architecture with differences in connecting loops

**Figure 1:**
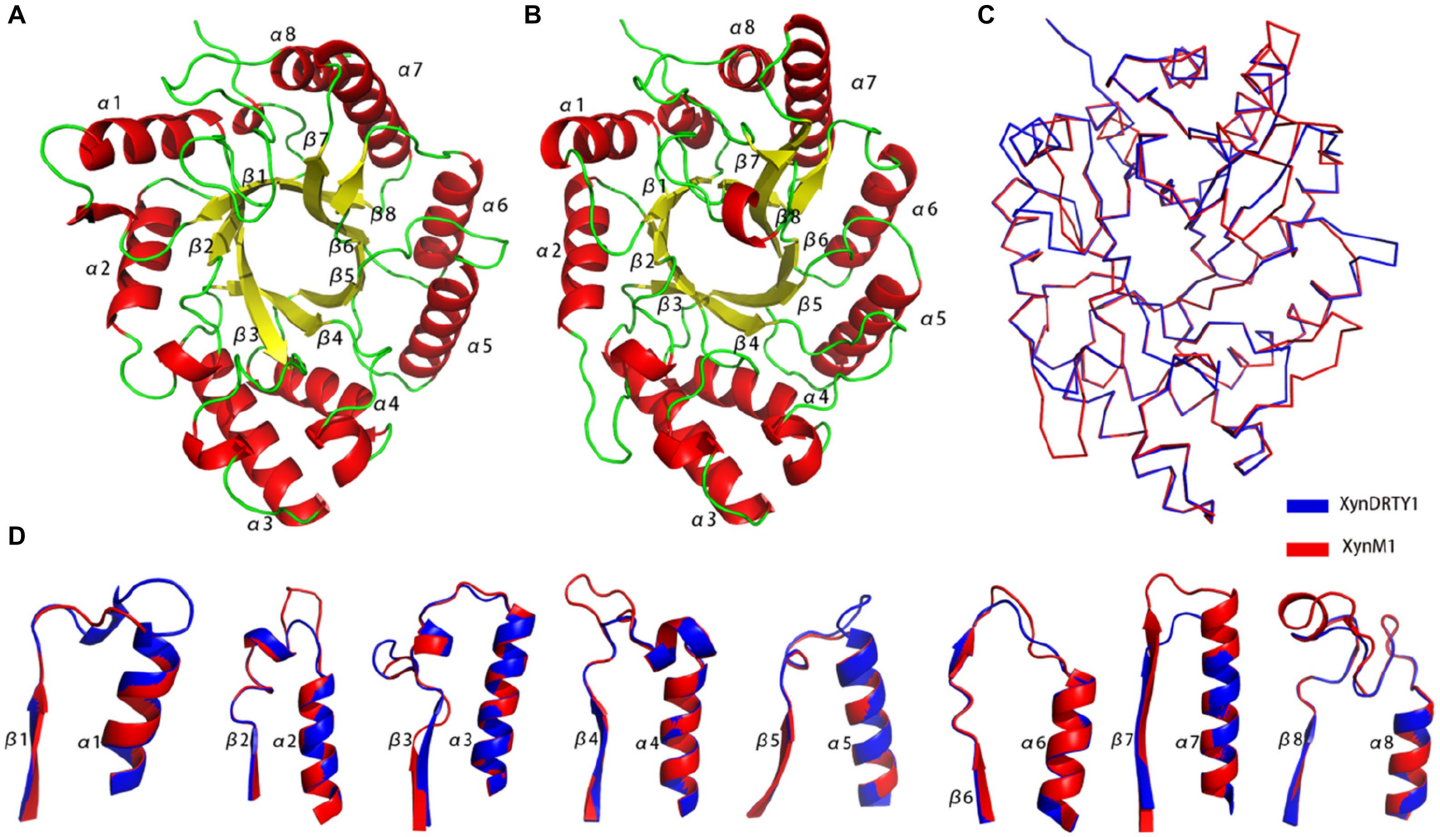
Structural representation and comparison of thermophilic xylanases. Cartoon representation of the structural models of the XynDRTY1 **(A)** and XynM1 **(B)** colored by the secondary structural elements. **(C)** The backbone superposition of the XynDRTY1 (blue) and XynM1 (red). **(D)** Comparison of connecting loops after the β-strand and before the α-helix in the XynDRTY1 (blue) and XynM1 (red).

**Figure 2:**
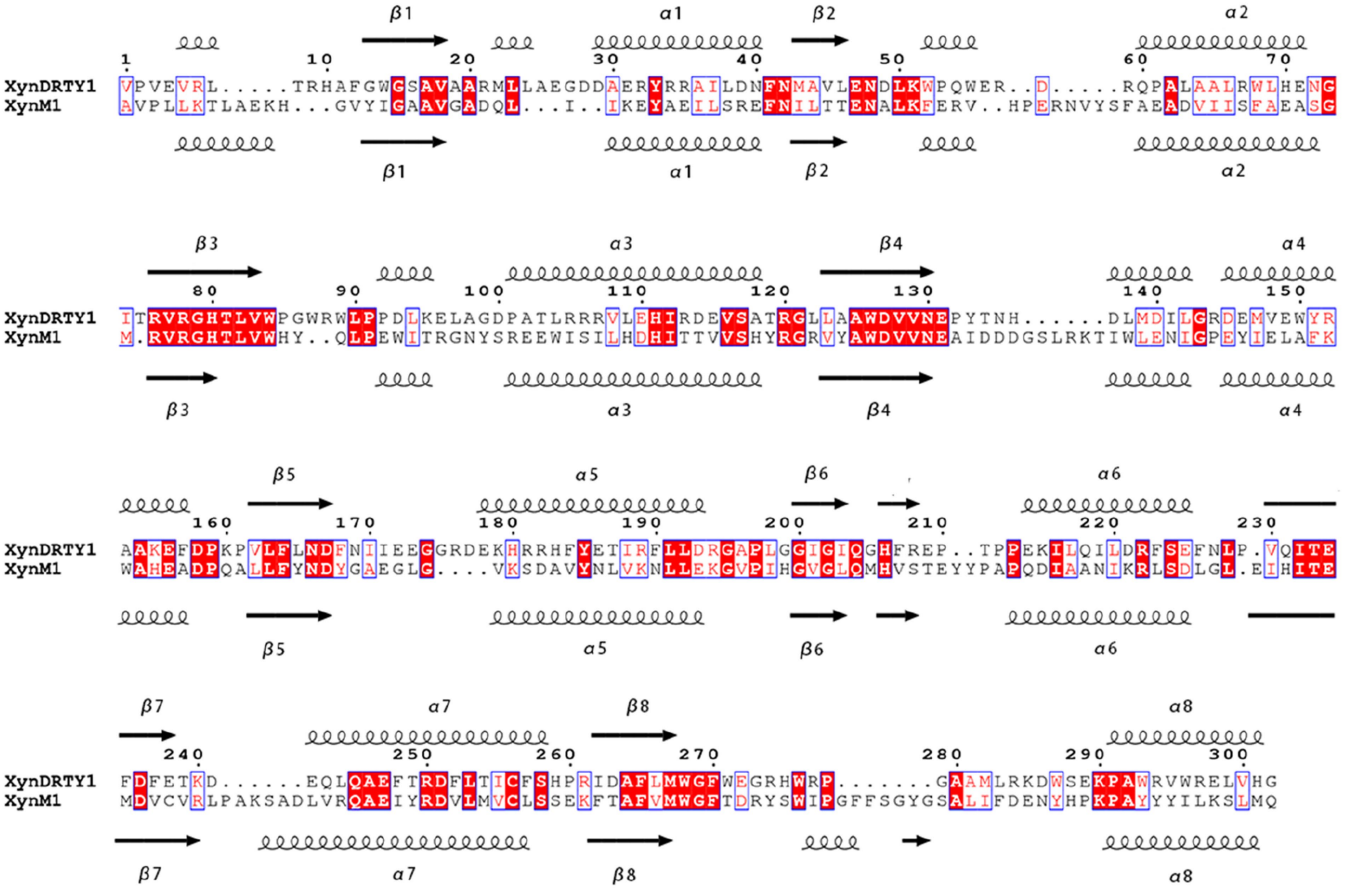
Structure-based sequence alignments of the XynDRTY1 and XynM1. Identical or similar residues are framed by blue lines, where identical residues are white on a red background and similar residues are red on a white background. The regular secondary structural elements were sequentially numbered and represented as spirals and arrows for α helices and β strands, respectively.

The overall structures of the XynDRTY1 and XynM1 exhibit a typical “salad-bowl” shape which consists of eight pairs of β-strand and α-helix to form a barrel core and a peripheral spiral-like wrapping, respectively (Figure 1). This structural architecture is commonly referred to as the TIM-barrel fold because it was first found in the triosephosphate isomerase (TIM) (Wierenga, 2001). These β-strands and α-helices were numbered sequentially from the N-terminus as β1–β8 and α1–α8, respectively. There are two cases of the connecting loops located between the α-helix and the β-strand. One is after the β-strand before the α-helix (called βα_loop) and the other is after α-helix before β-strand (called αβ_loop). Among those connecting loops, the βα_loop3, together with βα_loop 7 and 8 (Figure 1D), constitutes an extended deep cleft on the surface for substrate binding. By comparing the sequence alignments of the XynDRTY1 and XynM1 (Figure 2), it is more obvious that the βα_loop is relatively long and variable in insertion or deletion while the αβ_loop is very short and conservative.

The structural superimposition of these two thermophilic xylanases yielded the RMSD values of 1.76 Å with only 35.86% sequence identity, suggesting that the TIM-barrel fold has a stable structural architecture and tolerates diverse amino acid combinations to accommodate different biological selection pressures. Except for the structural constraints of the TIM-barrel fold, the structural differences between the XynDRTY1 and XynM1 are mainly concentrated in the βα_loops (Figure 1D). By partially aligning these loops, it can be seen that the βα_loops, especially βα_loop1, 2, 4, 5, 7, and 8, exhibit different structural orientations in these two thermophilic xylanases despite the highly conserved α-helices and the β-strands are structurally conserved in the TIM-barrel fold.

Although the structure of an enzyme can largely help decipher its biological and catalytic function, the molecular dynamics of the enzyme structure is an important factor to be considered in distinguishing the catalytic properties, especially the thermodynamic stability. Indeed, the dynamic, kinetic, and thermodynamic properties of a protein define its conformational states and the key structural determinants for its function. Therefore, thermophilic xylanases from hot springs were investigated by MD simulations in this study to reveal the contribution of their structural and dynamic determinants to the high-temperature adaptation.

### 3.2. Structural dynamics and thermal stability

**Figure 3:**
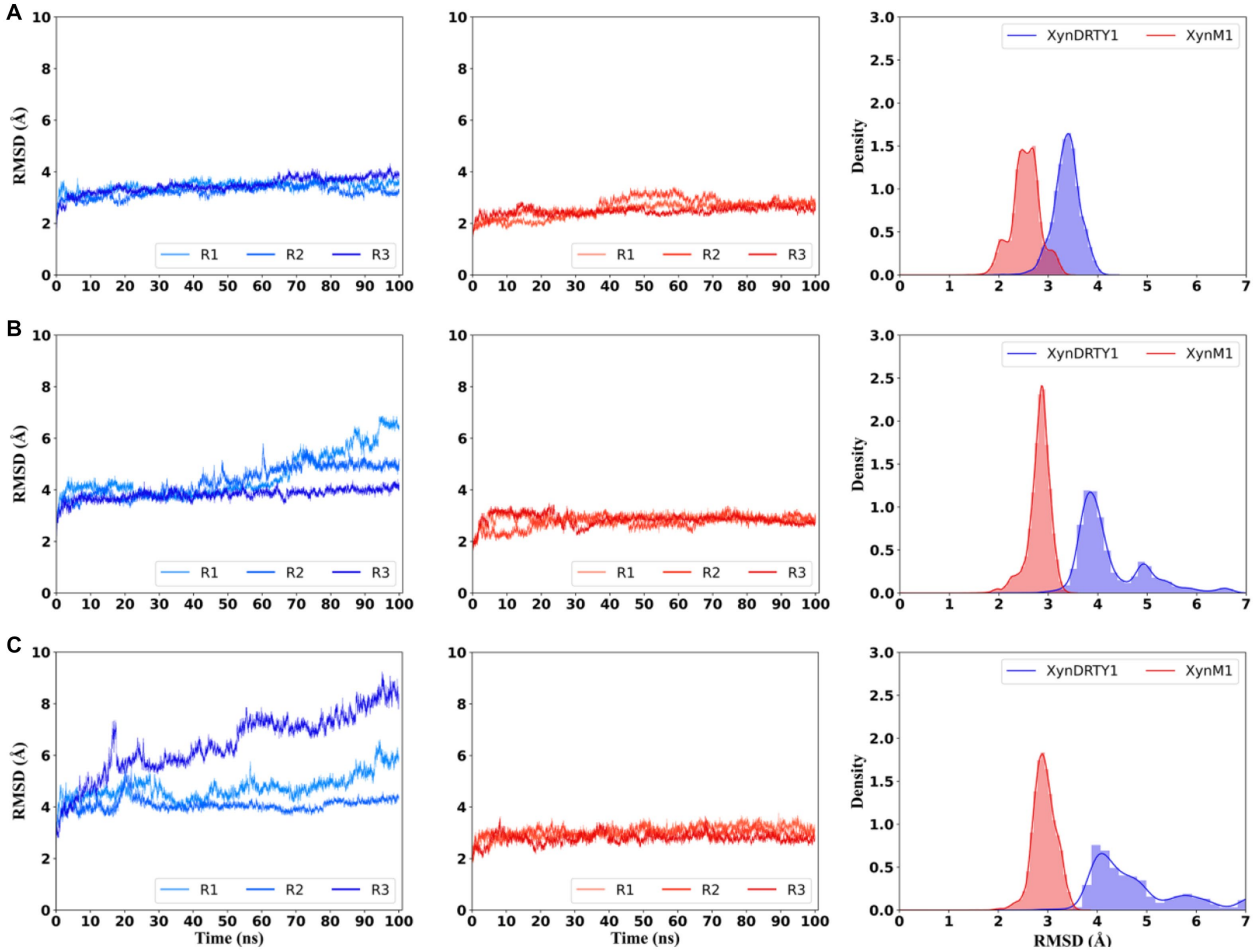
Temporal evolution of backbone root means square deviation (RMSD) values and their histogram of the XynDRTY1 (blue lines) and XynM1 (red lines) with respect to the corresponding initial structure at 300 K **(A)**, 350 K **(B)**, and 400 K **(C)**. The RMSD values of three replicas (R1-3) for each thermophilic xylanase were shown in different colors. All replicas were concatenated to calculate histogram statistics (right side) of RMSD values at each temperature, representing the distribution density of different RMSD values.

The structural dynamics and thermal stability of the XynDRTY1 and XynM1 were evaluated by monitoring the temporal evolution of backbone RMSD values relative to the starting structures during the MD simulation at different temperatures (Figure 3). Although the structural models of both thermophilic xylanases are very similar, the XynDRTY1 was characterized by slightly larger structural deviations than the XynM1 at 300 K. From the histogram statistics, it can be seen that the RMSD values of the XynM1 and XynDRTY1 were mainly distributed between 2 to 3 Å and 3 to 4 Å, respectively. The subtle difference in RMSD at 300 K still revealed that the XynDRTY1 experienced a slightly larger structural deviation from the starting conformation than the XynM1, implying that the former has a lower structural stability and a poorer capability to tolerate the high-temperature environment than the latter.

As temperatures increased, significantly larger dynamics in RMSD were observed for the XynDRTY1 while the XynM1 remained more stable. For the XynDRTY1, an increased trend of RMSD values was started from about 50 ns and 15 ns at 350 K and 400 K, respectively, indicating that the structure of the XynDRTY1 is susceptible to temperature and exhibits a low tolerance to the high temperature. In contrast, no significant RMSD fluctuations were observed for the XynM1 in all simulation temperatures, revealing the XynM1 has a very stable structure and a large high-temperature adaptation. The increasing temperature made the XynDRTY1 show a significant migration of the RMSD value distribution from a single peak between 3 and 4 Å at 300 K, to double peaks in 4 and 5 Å, respectively at 350 K, and finally to a broad distribution range from 4 to 6 Å. In contrast, the RMSD distribution of the XynM1 was only constrained around 3 Å. The difference in the distribution of RMSD values indicated that the XynDRTY1 has larger structural fluctuations and poorer thermal stability than the XynM1. The RMSD density plot showed that these two thermophilic xylanases shifted to a larger RMSD distribution as the temperature increased. At the same temperature, the RMSD values of the XynM1 were concentrated in a smaller region compared to the XynDRTY1, indicating its thermal stability. The RMSD values of the XynDRTY1 were always located at a larger level and its distribution was gradually dispersed with the temperature increasing, revealing that the XynDRTY1 is highly influenced by temperature.

By comparing the structural deviation of these two thermophilic xylanases, the structural stability, tight packing, and rigid folding possessed by the XynM1 appeared to contribute positively to thermal resistance. The above results suggested that stabilizing the overall structure of xylanases should be firstly considered to improve its high-temperature adaptability.

### 3.3. Conformational flexibility in different structural regions

**Figure 4:**
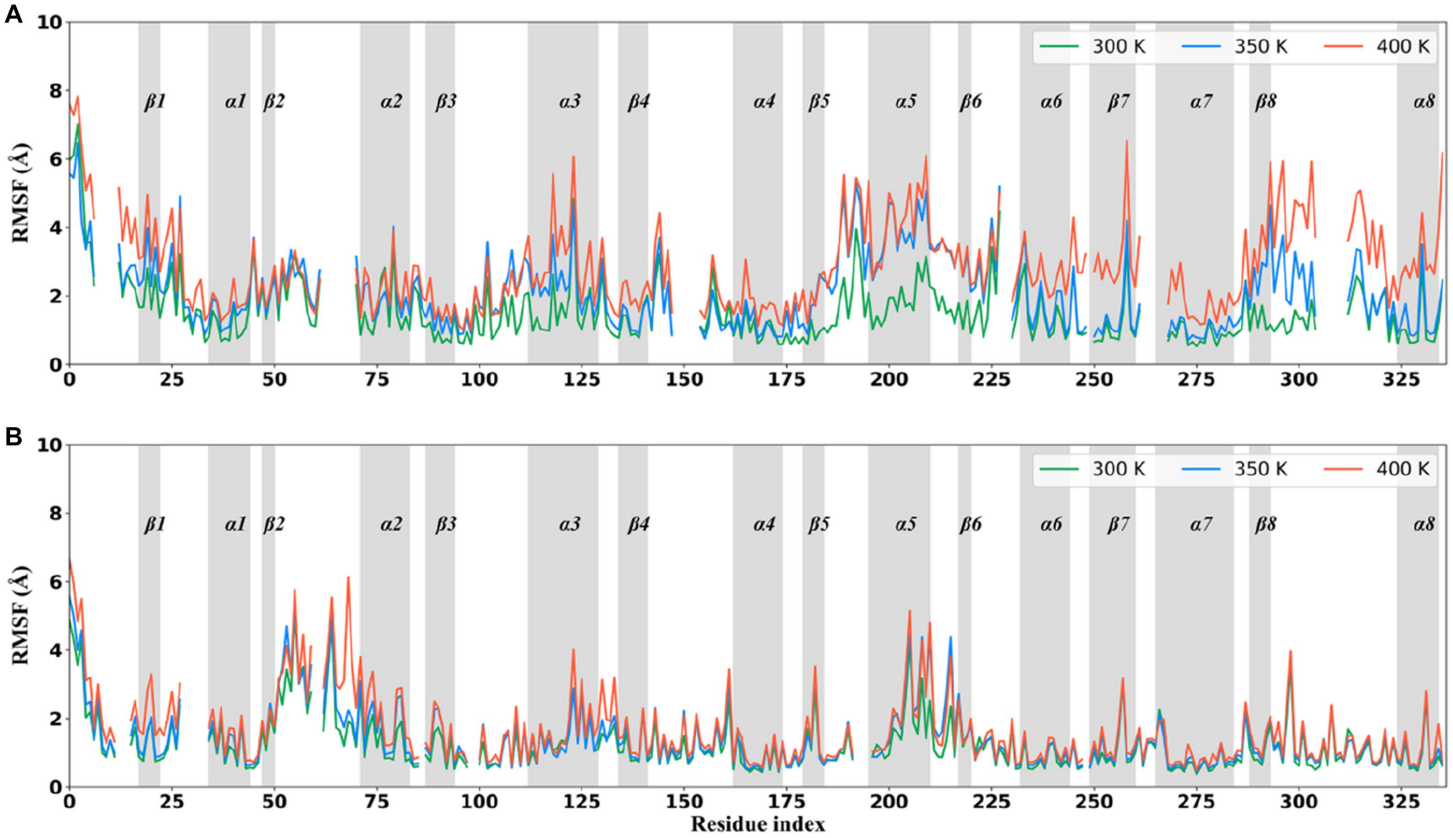
Per-residue C_α_ atom root mean square fluctuation (RMSF) curves averaged in three replicas of the XynDRTY1 **(A)** and XynM1 **(B)** during the molecular dynamics simulation at 300 K (green line), 350 K (blue line), and 400 K (red line). The residue index was according to the structure-based alignment of the XynDRTY1 and XynM1 (Figure 2).

To evaluate the conformational flexibility and high-temperature tolerance of these two thermophilic xylanases, per-residue RMSF on all C_α_ atoms was calculated and monitored during the simulations at different temperatures (Figure 4). The RMSF curves were discontinuous because they were shown at structurally equivalent positions obtained by superimposing the XynDRTY1 and XynM1. The same RMSF pattern, an expected increase in residue fluctuations with increasing temperature, can be observed in both thermophilic xylanases. Consistent with the previous structural dynamics, the XynDRTY1 generally had larger RMSF values than the XynM1 at the same temperature, revealing that the former exhibited higher conformational flexibility than the latter.

As the temperature increased, a few regions with significantly increased RMSF values were found. Except for the N/C-terminus, the region centered on β1, the tail of βα_loop3, α3, βα_loop5, α5, β6, βα_loop6, α6, β7, α7, β8, βα_loop8, and α8 in the XynDRTY1 experienced significant conformational flexibility enhancement with increasing temperature. Among the above regions, the β1, βα_loop3, α6, β8, βα_loop8, and α8 had larger RMSF values only at 400 K, indicating that these regions are more tolerant to temperature and require relatively high temperatures to significantly increase their conformational flexibility. For βα_loop5, α5, β6, and βα_loop6, temperature conditions at 350 and 400 K made these regions exhibit a similarly higher RMSF level, suggesting that they are sensitive to high temperatures. Only α3 appeared to distribute its RMSF values evenly at temperatures between 300 and 400 K, exhibiting moderate conformational flexibility compared to the highly flexible regions mentioned above and other conserved regions. In the case of the XynM1, only regions such as β1 and the tail of βα_loop2 had a limited RMSF difference at high-temperature conditions. The overall RMSF distribution of the XynM1 was almost unaffected by high temperature, demonstrating its restricted conformational flexibility and high-temperature tolerance.

### 3.4. Conformational populations

**Figure 5:**
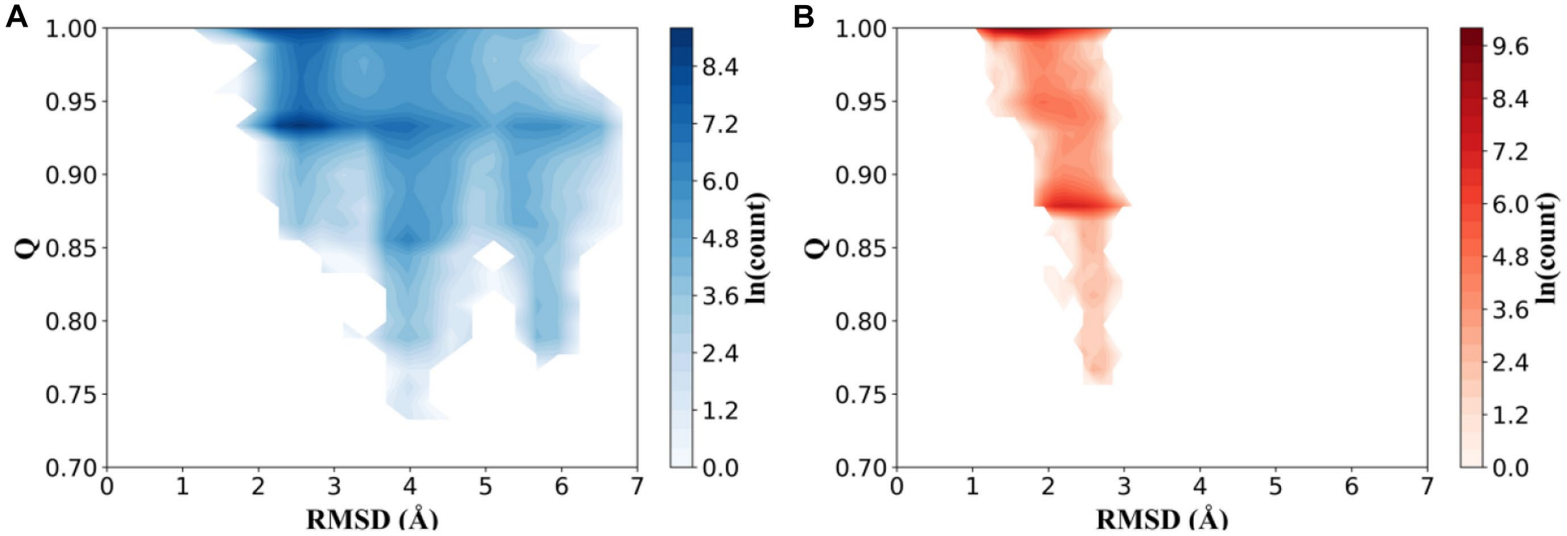
Conformational distributions of the XynDRTY1 **(A)** and XynM1 **(B)** as a function of the root means square deviation (RMSD) and the fraction of native contacts (Q). The color bar represents the logarithm (ln) of the count.

To compare the effect of high-temperature influence on the conformational distribution of thermophilic xylanases, the trajectories of the XynDRTY1 and XynM1 were projected into a space consisting of the RMSD describing the structural dynamics and the Q representing the unfolding degree. The fraction of native contacts (Q) had been used as determining protein folding mechanisms in atomistic simulations (Best et al., 2013). Although neither of the two thermophilic xylanases exhibited significant unfolding during the high-temperature simulation (both had Q values predominantly greater than 0.8), the distribution of their natural contact content was different (Supplementary Figure S7). As shown in Figure 5, conformations of the XynDRTY1 were distributed in the region with RMSD values from 1 to 7 Å and Q from 0.7 to 1, while the XynM1 was confined to the RMSD region from 1 to 3 Å during the simulation. The large range of conformational distribution indicates that the XynDRTY1 has a large conformational diversity than the XynM1, implying the XynDRTY1 exhibits a weak thermal resistance under the influence of high temperature.

### 3.5. Disintegration of regular secondary structures

**Figure 6:**
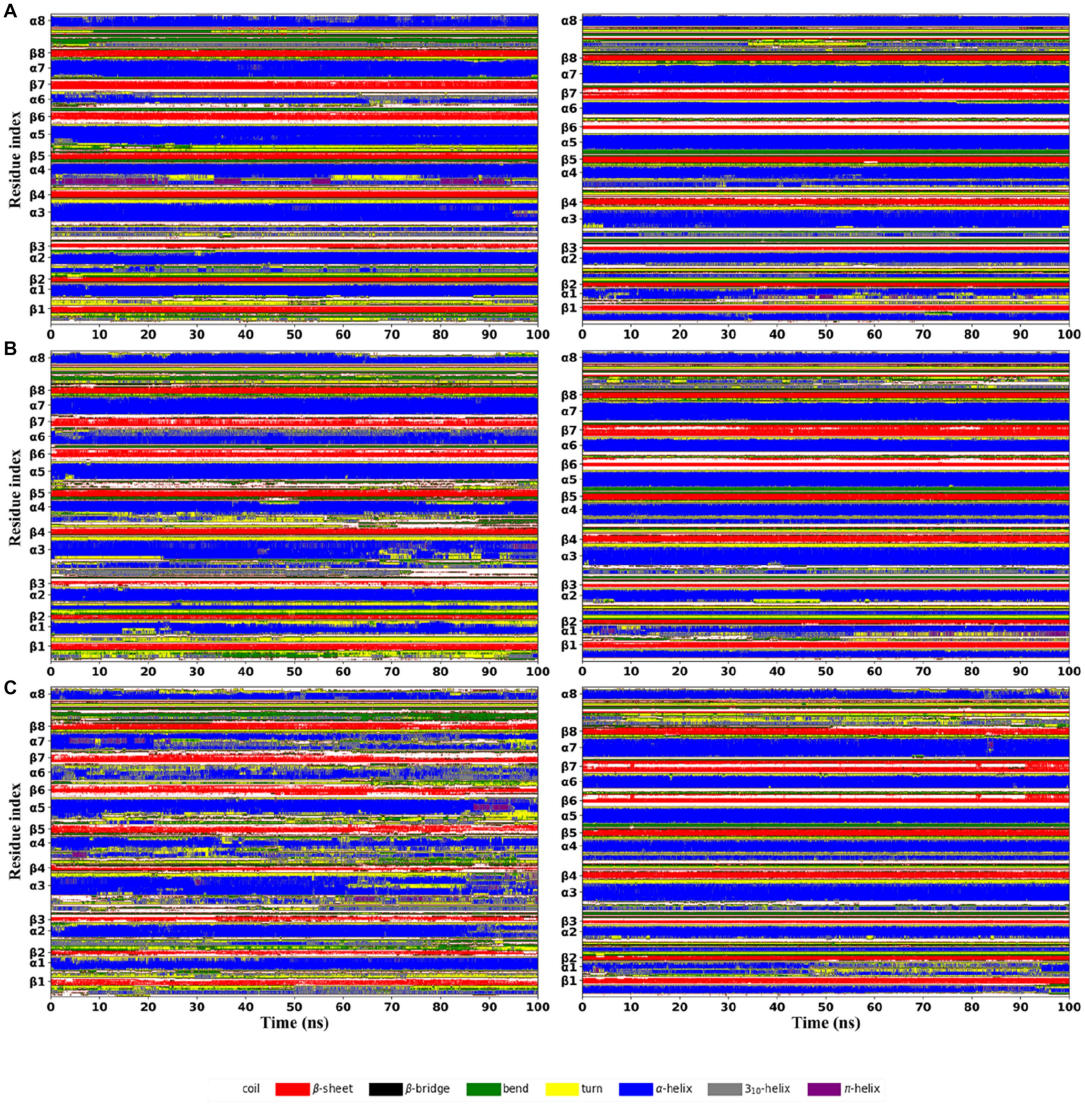
Time evolution of secondary structure propensities of the XynDRTY1 (left) and XynM1 (right) from the first replica of the molecular dynamics simulation at 300 K **(A)**, 350 K **(B)**, and 400 K **(C)**. The residue position of the regular secondary structures in the corresponding initial structure was marked along the vertical coordinate. Different secondary structure types were shown in different colors.

The per-residue secondary structure assignment as a function of simulation time was used to measure the effect of increasing temperature on the local structural environments (Figure 6). At 300 K, both thermophilic xylanases maintained the specific folding of eight β-strands and most of α-helix except α6 in the TIM-barrel fold during the simulation. A few segments of α6 in the XynDRTY1 evolved into the turn secondary structure element after about 70 ns, while the α6 of the XynM1 remained as the starting. Comparison of α6 secondary structure suggests that it might be the initiating factor for the thermodynamic differences between the XynDRTY1 and XynM1. There were more turn and coil secondary structure elements in the XynDRTY1 than in the XynM1, especially in the N-terminus, βα_loop1, 3, 4, 5, and 7, implying that these regions will disintegrate at high-temperature conditions.

As the temperature increases, the regular secondary structures in the XynDRTY1 drastically lost/changed, while the distribution of the secondary structures in the XynM1 remained unchanged. At 350 K, careful comparisons revealed that minor losses/changes in secondary structural elements were observed in the α1, β3, α3, α4, β6, and β7 of the XynDRTY1. When the structure was heavily denatured at 400 K, most of the lost regular secondary structures became bends, turns, and coils, making green, yellow, and white the dominant color, especially for the β2-7, α3-4, and α5. For the XynM1, only α1 exhibited disordered at 400 K.

### 3.6. Survival probabilities of residue contacts

**Figure 7:**
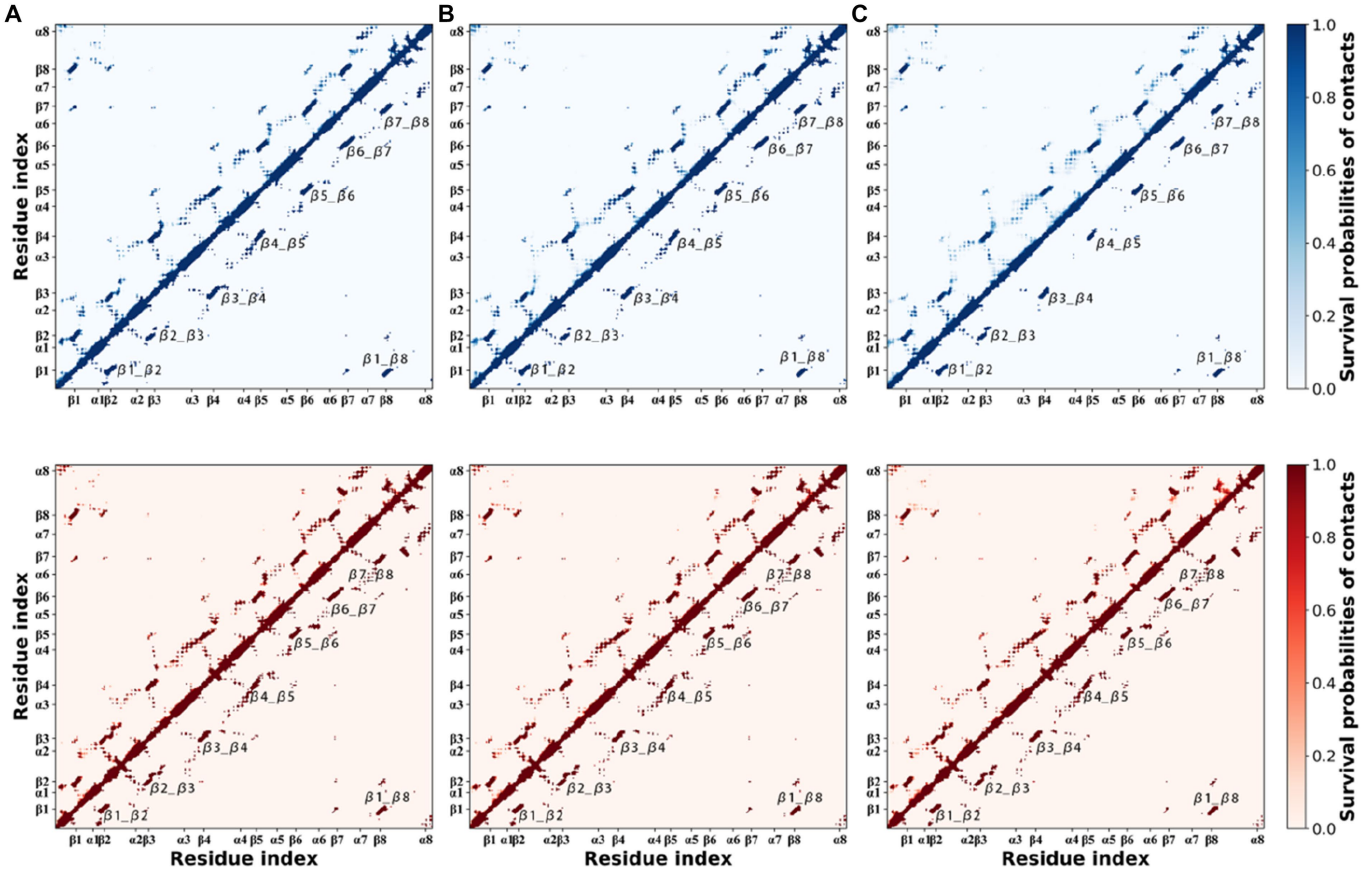
Survival probabilities of residue contacts in the XynDRTY1 (blues) and XynM1 (reds) at 300 K **(A)**, 350 K **(B)**, and 400 K **(C)**. The upper triangle shows all occurring contacts. The lower triangle only shows those contacts with a probability greater than 0.8.

In MD simulations, high temperatures force certain unstable residue contacts to break as the temperature increases. For each thermophilic xylanase at the same temperature, 200 snapshots of MD trajectory were extracted from each replica at 0.5 ns intervals. Every snapshot was used to construct a residue contact map where pairwise residues whose C_α_ atom distance was less than 10 Å were identified. For three replicas of each thermophilic xylanase at the same temperature, all residue contact maps were calculated as survival probabilities of residue contacts (Figure 7). The survival probability of the residue contact characterizes the duration ratio of residue contact during the simulation, with a higher probability representing a longer residue contact duration. To highlight stable residue contacts, residue contacts with a survival probability greater than 0.8 were shown in the lower triangle of Figure 7.

Looking at the probabilities of residue contacts, the XynDRTY1 and XynM1 have similar patterns of residue contact composition in general. In addition to the residue contacts determined by the backbone atoms (diagonal line in Figure 7), inter-β-strand contacts, labeled as β1_β2, β2_β3, β3_β4, β4_β5, β5_β6, β6_β7, β7_β8, and β1_β8, between eight β-strands existed stably during MD simulations. At 300 K, the difference in residue contact duration between the XynDRTY1 and XynM1 mainly involved the local regions around the inter-β-strand contacts. The XynM1 has more stable contacts surrounding the inter-β-strand contacts than the XynDRTY1. With increasing temperature, most of the residue contacts around the inter-β-strand contacts were gradually lost in both thermophilic xylanases. Especially in the XynDRTY1, these surrounding contacts and even some of the residue contacts between eight β-strands were disintegrated. These results implied that a tight package for eight β-strands is crucial for the thermal stability of xylanase.

### 3.7. Coevolutionary information from the xylanase family

**Figure 8:**
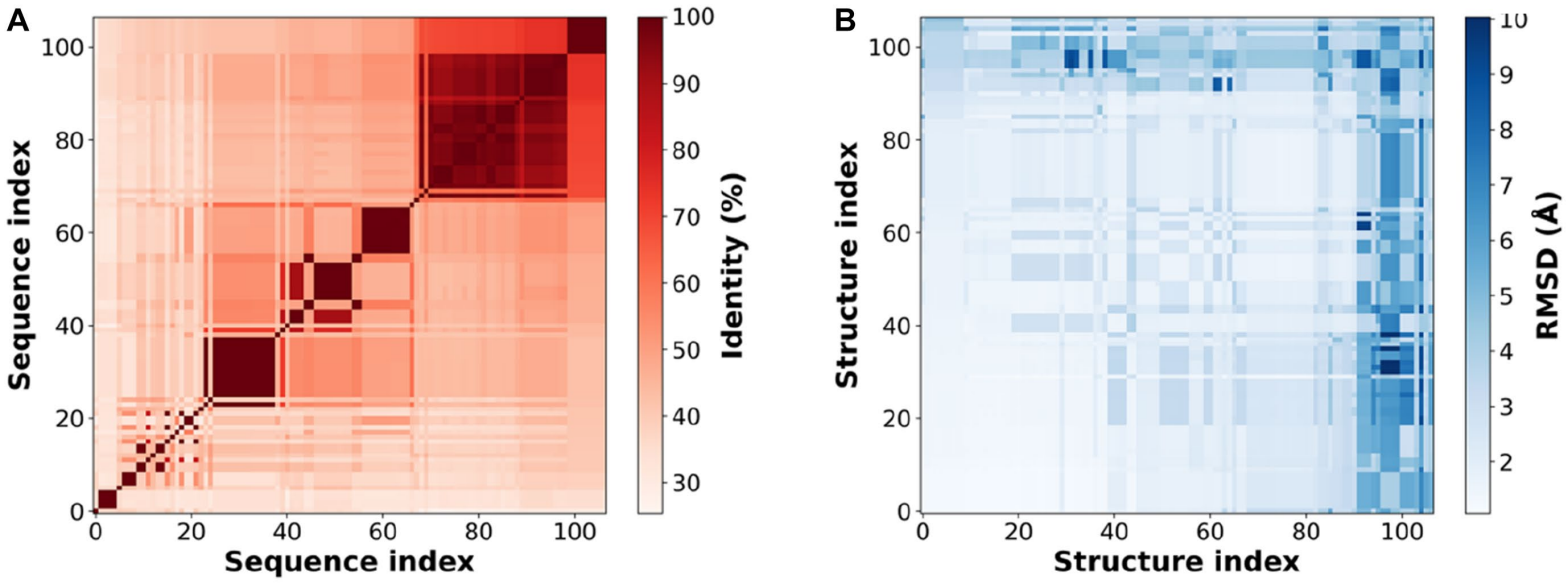
Sequence identity **(A)** and structural similarity **(B)** of experimental resolved xylanase structures. Sequence or structure index is the serial number of 105 experimentally resolved structures of xylanases (PDB IDs listed in Supplementary Table S1).

Consistent with the original reports of the XynDRTY1 and XynM1, our sequence analysis and structural modeling indicated that these two thermophilic xylanases belong to the Glycoside Hydrolase family 10 (GH-10). To analyze the high-temperature adaptation of thermophilic xylanases at the enzyme family level, 105 experimentally resolved structures of xylanase were extracted from the GH10 family. Sequence and structural alignments of these xylanase structures (Figure 8) demonstrated that the xylanase family has a low level of sequence identity (mostly <60%) but shares a very similar structural conformation with pairwise RMSD values generally less than 5 Å.

**Figure 9:**
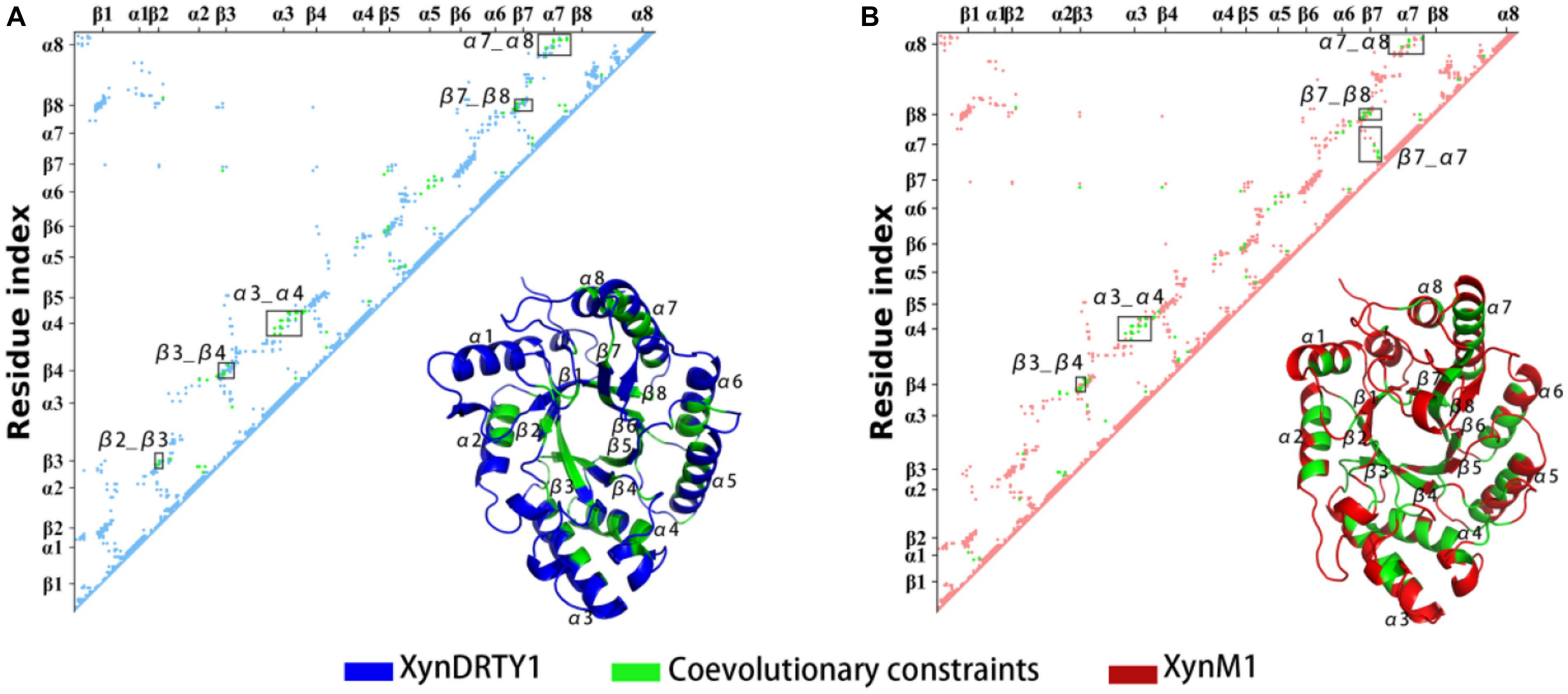
Coevolutionary constraints from the xylanase family and its mapping in the structures of the XynDRTY1 **(A)** and XynM1 **(B)**. In the residue matrix, the contacts in the XynDRTY1 and XynM1 were colored blue and red, respectively. Coevolutionary constraints from the xylanase family were marked in green.

The above experimental resolved xylanase structures were used to generate multiple sequence alignments (MAS) and subsequently to obtain coevolutionary information. The coevolutionary constraints from the xylanase family were mapped into the structures of the XynDRTY1 and XynM1 (Figure 9). These coevolutionary constraints represented as residue contacts are mainly distributed in the β2_β3, β3_β4, α3_α4, β7_β8, and α7_α8 for the XynDRTY1, while β7_α7 also has coevolutionary constraints for the XynM1. These coevolutionary contacts together with the TIM-barrel fold jointly determine the structural basis of xylanase.

## 4. Conclusion

In this study, two thermophilic xylanases, the XynDRTY1 and XynM1, from hot-spring microorganisms were investigated by MD simulations and coevolutionary analysis to decipher the potential structural determinants underlying their high-temperature adaptation. Although the XynDRTY1 and XynM1 have a similar fold architecture, there are structural differences in βα_loops. Comparative analysis of MD trajectories revealed that the XynM1 exhibits smaller structural dynamics and larger thermal stability than the XynDRTY1. The conformational flexibility of the local regions indicated that these two thermophilic xylanases have different thermal resistance and some of the regular secondary structures also exhibited different disintegration patterns during temperature increases. These local regions are closely related to the high-temperature adaptation of xylanase, implying that stabilization of these regions is a feasible strategy to enhance the thermal stability of xylanase. Coevolutionary information from the xylanase family further specified the structural basis of xylanases. In conclusion, our study not only deciphered structural determinants underlying the high-temperature adaptation of thermophilic xylanase from hot-spring microorganisms but also provided direct help to improve the thermal stability of xylanase.

## Data availability statement

The datasets presented in this study can be found in online repositories. The names of the repository/repositories and accession number(s) can be found in the article/Supplementary material.

## Author contributions

YL conceived the study and wrote the manuscript. H-QP performed the simulations, analyzed the data, and plotted the graphs. L-QY reviewed and revised the manuscript. All authors contributed to the article and approved the submitted version.

## Funding

This work was supported by the National Natural Science Foundation of China (Grants Nos. 62241602 and 31960198) and the Open Research Program of State Key Laboratory for Conservation and Utilization of Bio-Resource in Yunnan (Grants No. 2021KF011).

## Conflict of interest

The authors declare that the research was conducted in the absence of any commercial or financial relationships that could be construed as a potential conflict of interest.

## Publisher’s note

All claims expressed in this article are solely those of the authors and do not necessarily represent those of their affiliated organizations, or those of the publisher, the editors and the reviewers. Any product that may be evaluated in this article, or claim that may be made by its manufacturer, is not guaranteed or endorsed by the publisher.

## Footnotes

^1^https://hits-mbm.github.io/conan

^2^https://www.ebi.ac.uk/interpro
