# Supplementary material for: Structural determinants underlying high-temperature adaptation of thermophilic xylanase from hot-spring microorganisms
Source: Front Microbiol. 2023 Jul 7;14:1210420. doi: 10.3389/fmicb.2023.1210420 (PMC10360402; doi:10.3389/fmicb.2023.1210420)
Supplement: Supplementary file 1 [file Data_Sheet_1.docx]

Supplementary Material

**Structural determinants underlying high-temperature adaptation of thermophilic xylanase from hot-spring microorganisms**

Yi Li^1,2,3,4^, Hong-Qian Peng^1^, and Li-Quan Yang^2,3*^

1. College of Mathematics and Computer Science, Dali University, Dali, China.

2. College of Agriculture and Biological Science, Dali University, Dali, China.

3. Key Laboratory of Bioinformatics and Computational Biology by Education Department of Yunnan Province, Dali University, Dali, China.

4. State Key Laboratory for Conservation and Utilization of Bio-Resource in Yunnan, Yunnan University, Kunming, China.

*Correspondence: Li-Quan Yang, [ylqbioinfo@gmail.com](mailto:ylqbioinfo@gmail.com).

**SUPPLEMENTARY TABLE S1** 105 experimentally resolved structures of the xylanase family.

| **PDB ID Chain ID** |
| --- |
| 1b30_A, 1b31_A, 1b3v_A, 1b3w_A, 1b3x_A, 1b3y_A, 1b3z_A, 1bg4_A, 1e0v_A, 1e0w_A, 1e0x_A, 1exp_A, 1fh7_A, 1fh8_A, 1fh9_A, 1fhd_A, 1gok_A, 1gom_A, 1goo_A, 1goq_A, 1gor_A, 1i1w_A, 1i1x_A, 1j01_A, 1k6a_A, 1nq6_A, 1od8_A, 1ta3_B, 1tux_A, 1ur1_A, 1v0k_A, 1v0l_A, 1v0m_A, 1v0n_A, 1v6y_A, 1vbr_A, 1vbu_A, 1xyz_A, 2bnj_A, 2dep_B, 2exo_A, 2g3i_A, 2g3j_A, 2g4f_A, 2his_A, 2xyl_A, 3cuf_A, 3cug_A, 3cuh_A, 3cui_A, 3cuj_A, 3emc_A, 3emq_A, 3emz_A, 3niy_B, 3nj3_B, 3nyd_A, 3o2l_A, 3wub_A, 3wue_A, 3wuf_A, 3wug_A, 4f8x_A, 4l4o_A, 4l4p_A, 4pmd_A, 4pmx_A, 4pmy_A, 4pmz_A, 4pn2_A, 4xv0_A, 4xx6_A, 5rg4_A, 5rg5_A, 5rg6_A, 5rg7_A, 5rg9_A, 5rga_A, 5rgc_A, 5rgd_A, 5rge_A, 5rgf_A, 5xzo_B, 5xzu_B, 5y3x_A, 6d5c_A, 6jdt_A, 6jdy_A, 6jdz_A, 6je0_A, 6je1_A, 6je2_A, 7k4q_B, 7k4r_A, 7k4s_A, 7k4t_A, 7k4u_A, 7k4x_A, 7k4y_A, 7k4z_A, 7nl2_B, 7wh6_A, 7wh7_A, 7wha_A, 7whe_A |


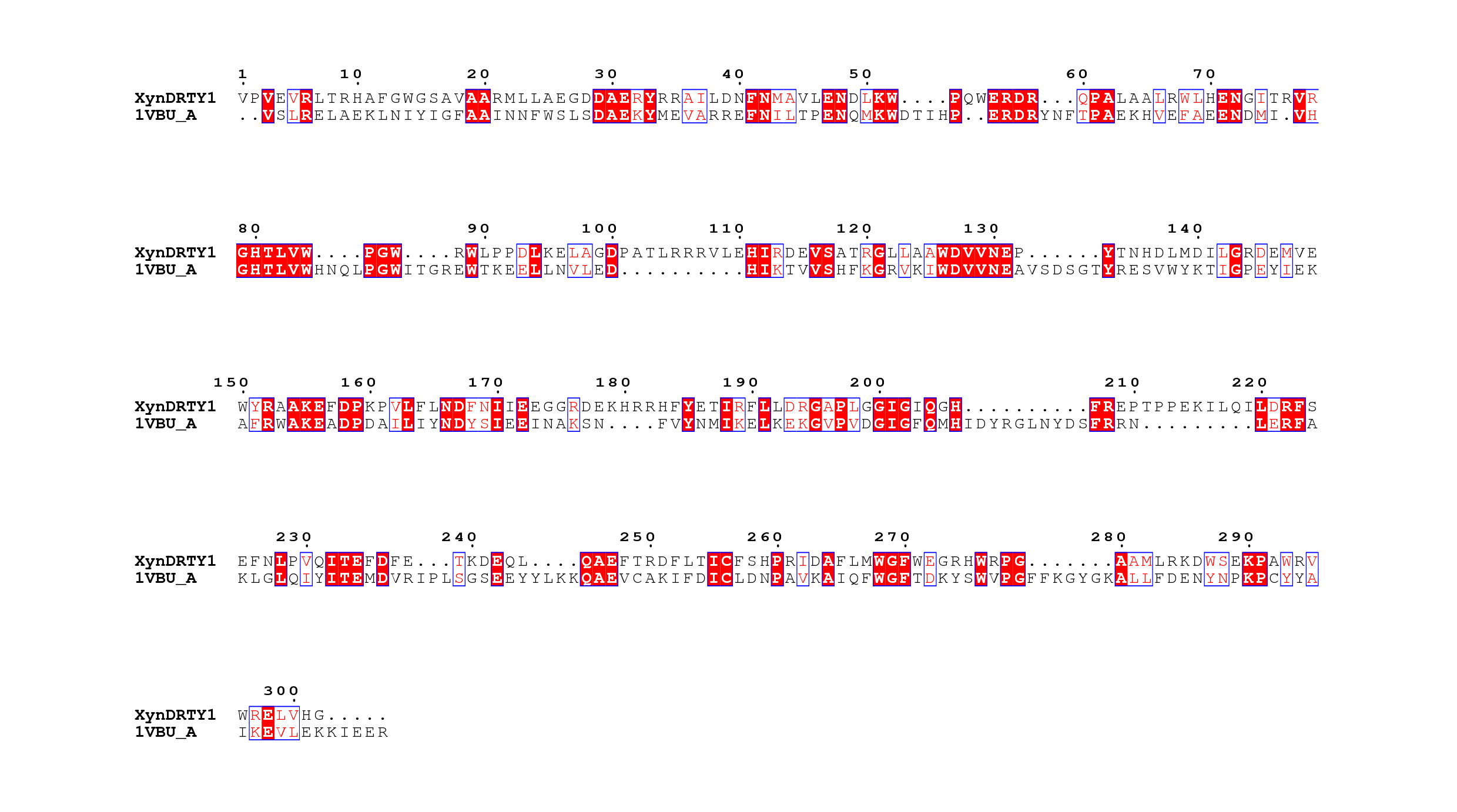


**SUPPLEMENTARY FIGURE S1**

Sequence alignments of the XynDRTY1 and reference structure (PDB ID: IVBU) which was also used by (Yin et al., 2022).


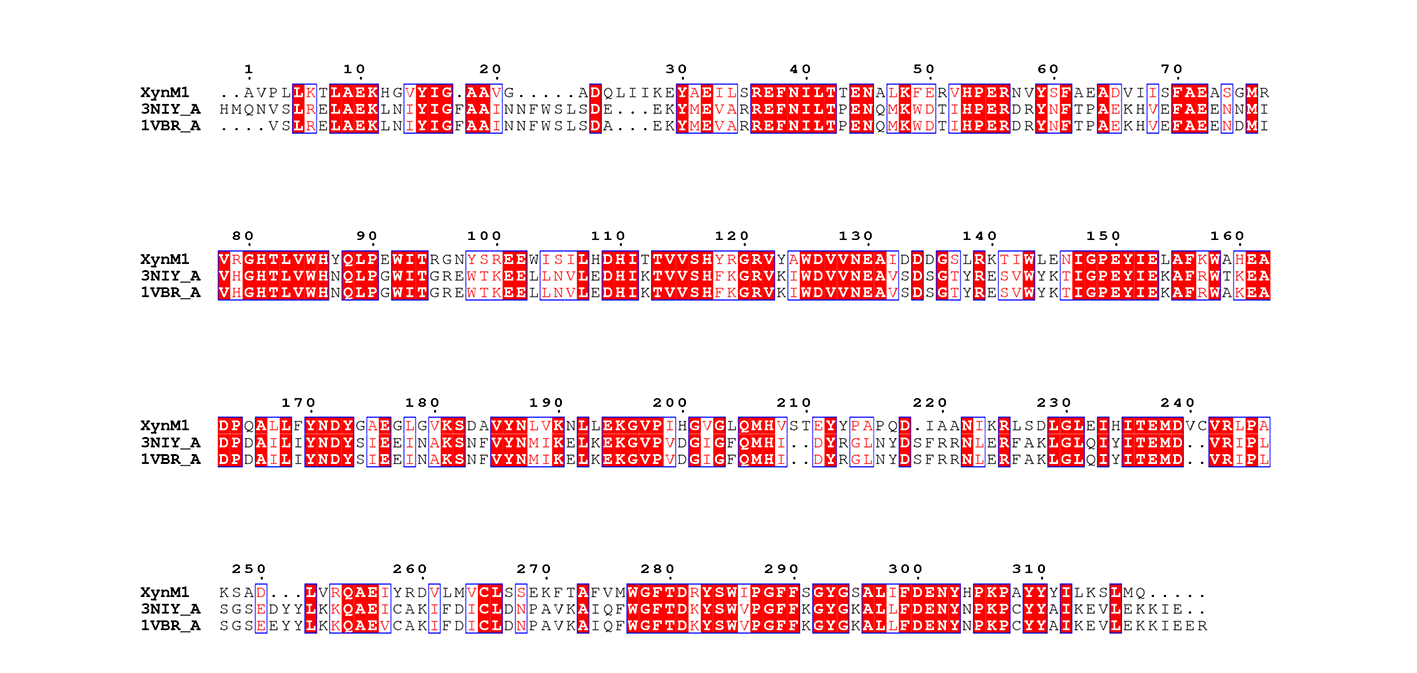


**SUPPLEMENTARY FIGURE S2**

Sequence alignments of the XynM1 and reference structure (PDB ID: 3NIY and IVBR) which were also used by (Joshi et al., 2020).


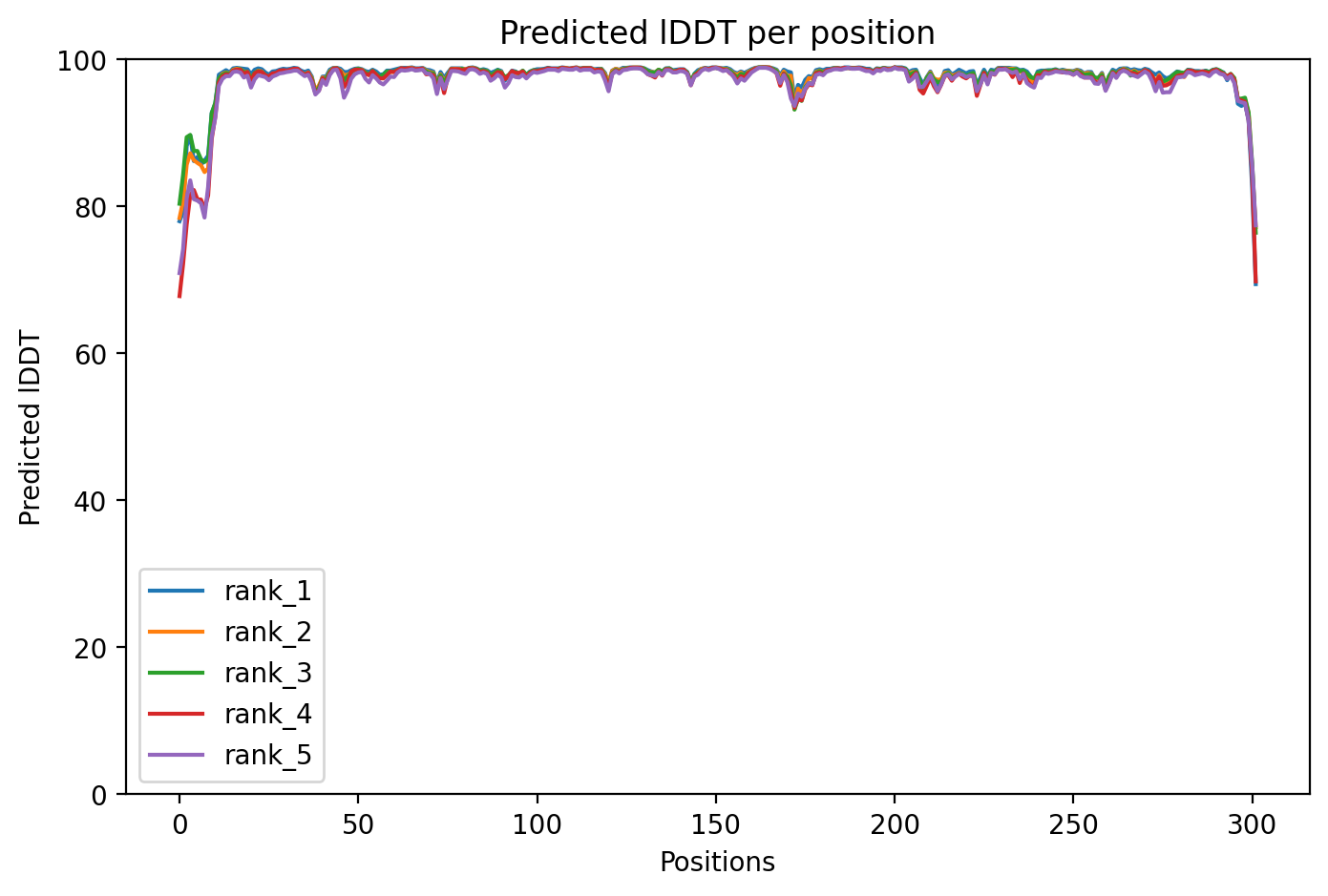


**SUPPLEMENTARY FIGURE S3**

Predicted Local Distance Difference Test (pLDDT) values of the XynDRTY1.


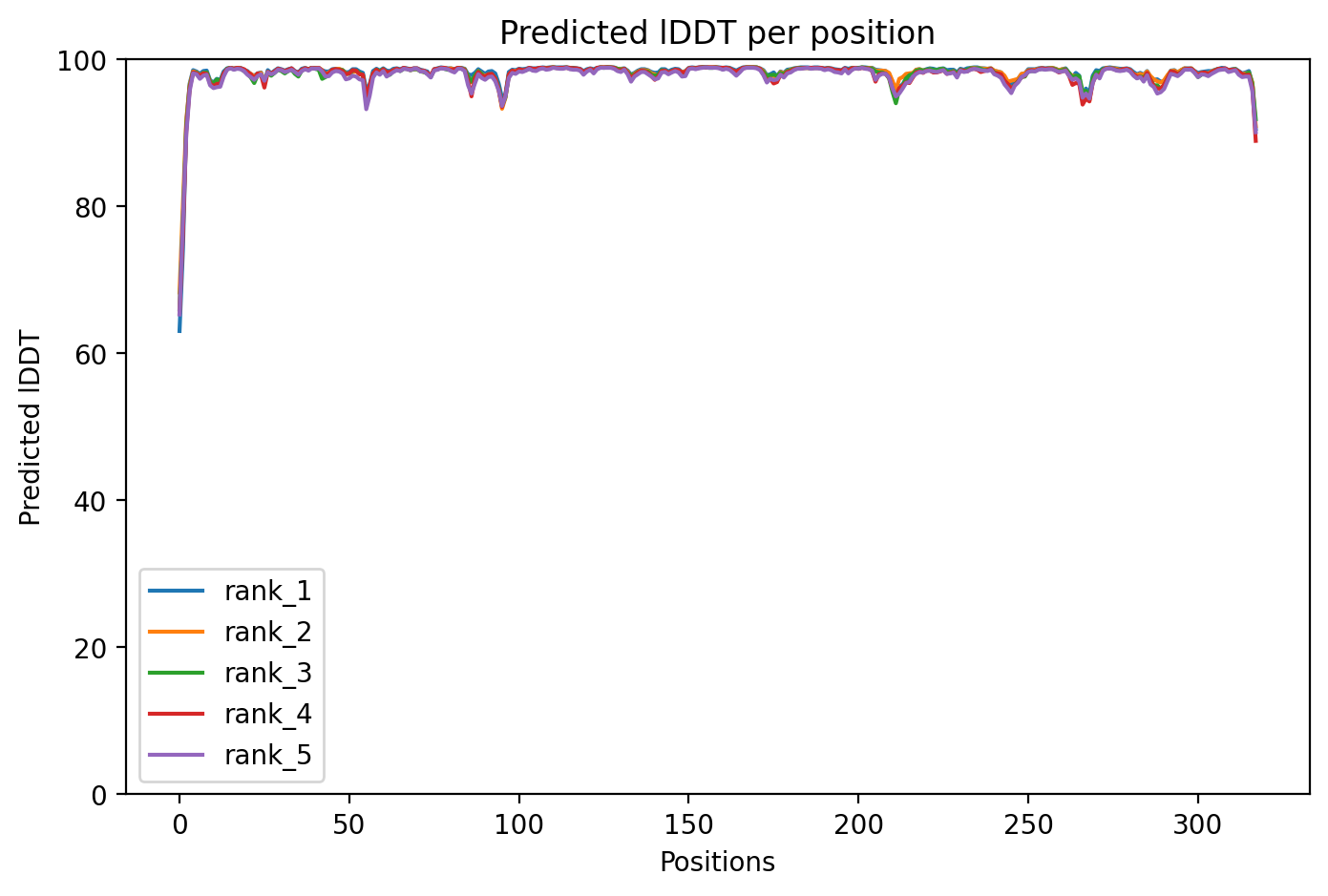


**SUPPLEMENTARY FIGURE S4**

Predicted Local Distance Difference Test (pLDDT) values of the XynM1.


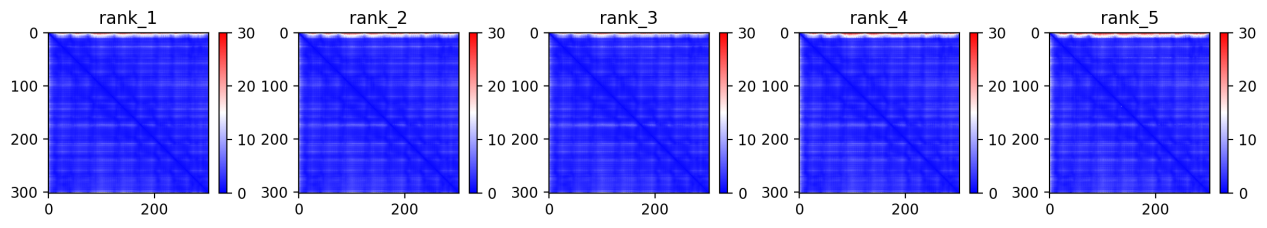


**SUPPLEMENTARY FIGURE S5**

Predicted Aligned Error (PAE) of the XynDRTY1.


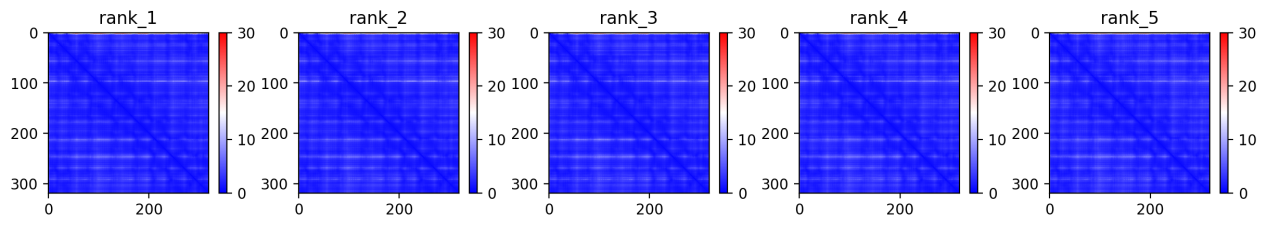


**SUPPLEMENTARY FIGURE S6**

Predicted Aligned Error (PAE) of the XynM1.


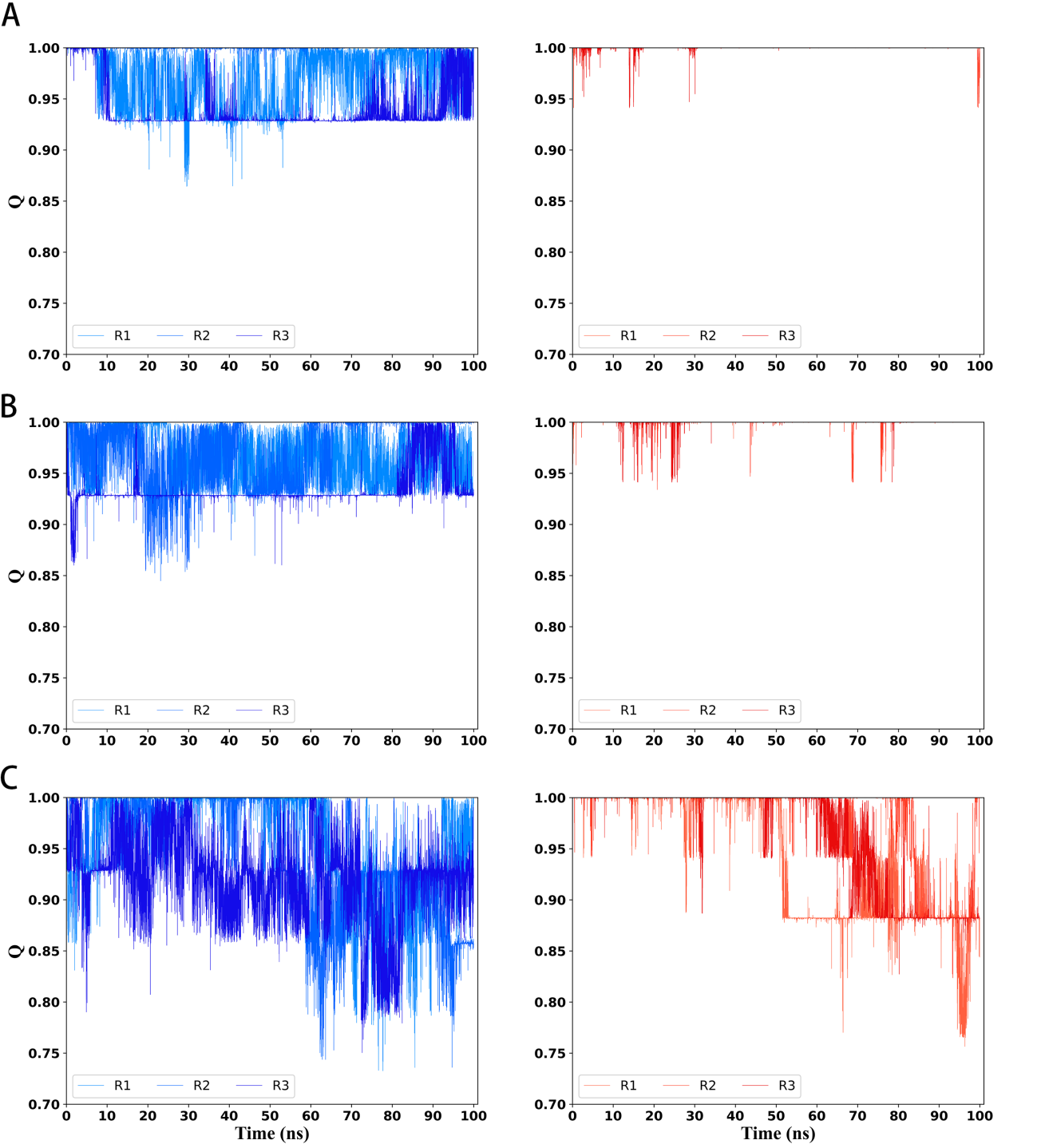


**SUPPLEMENTARY FIGURE S7**

Temporal evolution of the fraction of native contacts (Q) values from three replicas (R1-3) of the XynDRTY1 (blue lines) and XynM1 (red lines) at 300 K **(A)**, 350 K **(B)**, and 400 K **(C)**.


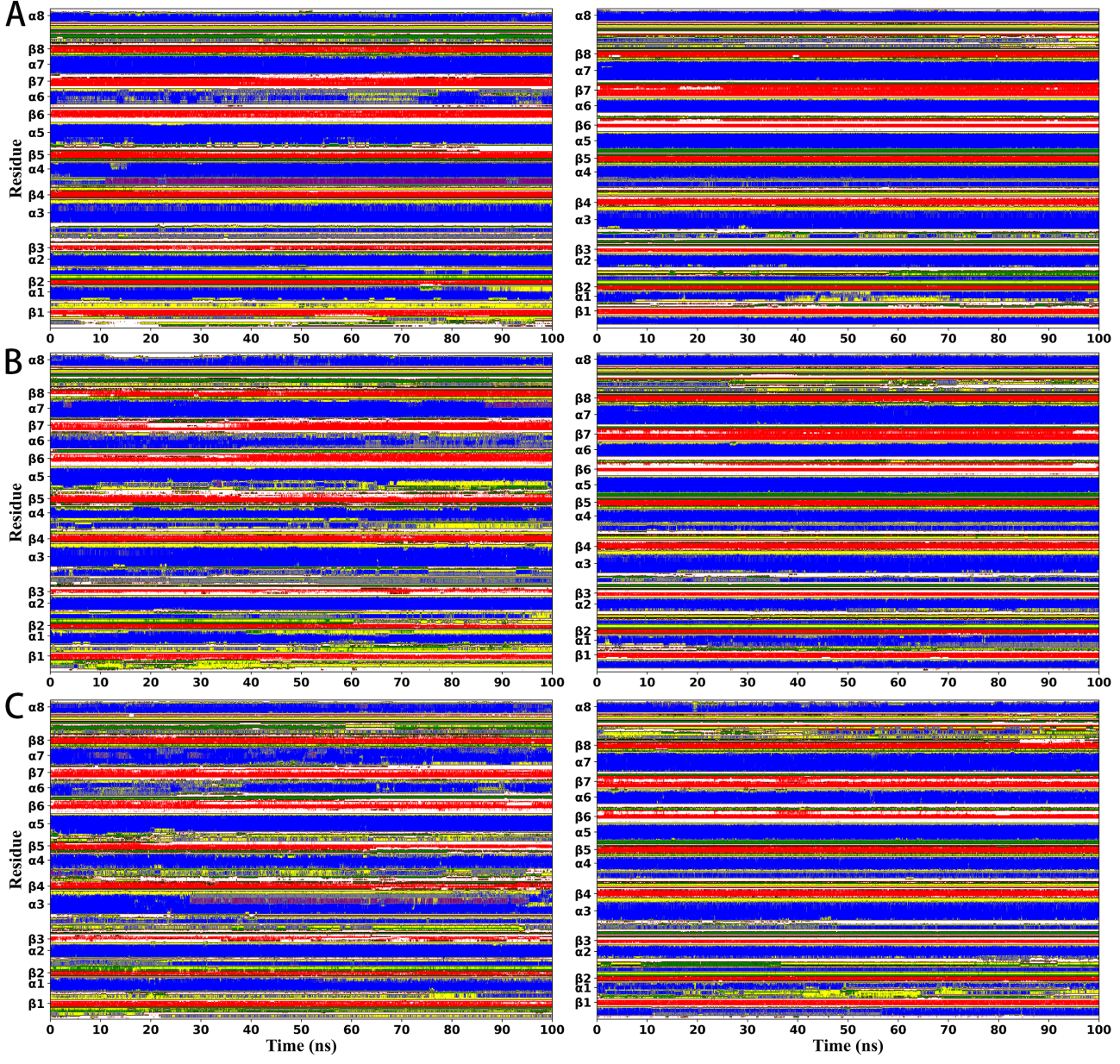


**SUPPLEMENTARY FIGURE S8**

Time evolution of secondary structure propensities of the XynDRTY1 (left) and XynM1 (right) from the second replica of the molecular dynamics simulation at 300 K **(A)**, 350 K **(B)**, and 400 K **(C)**.


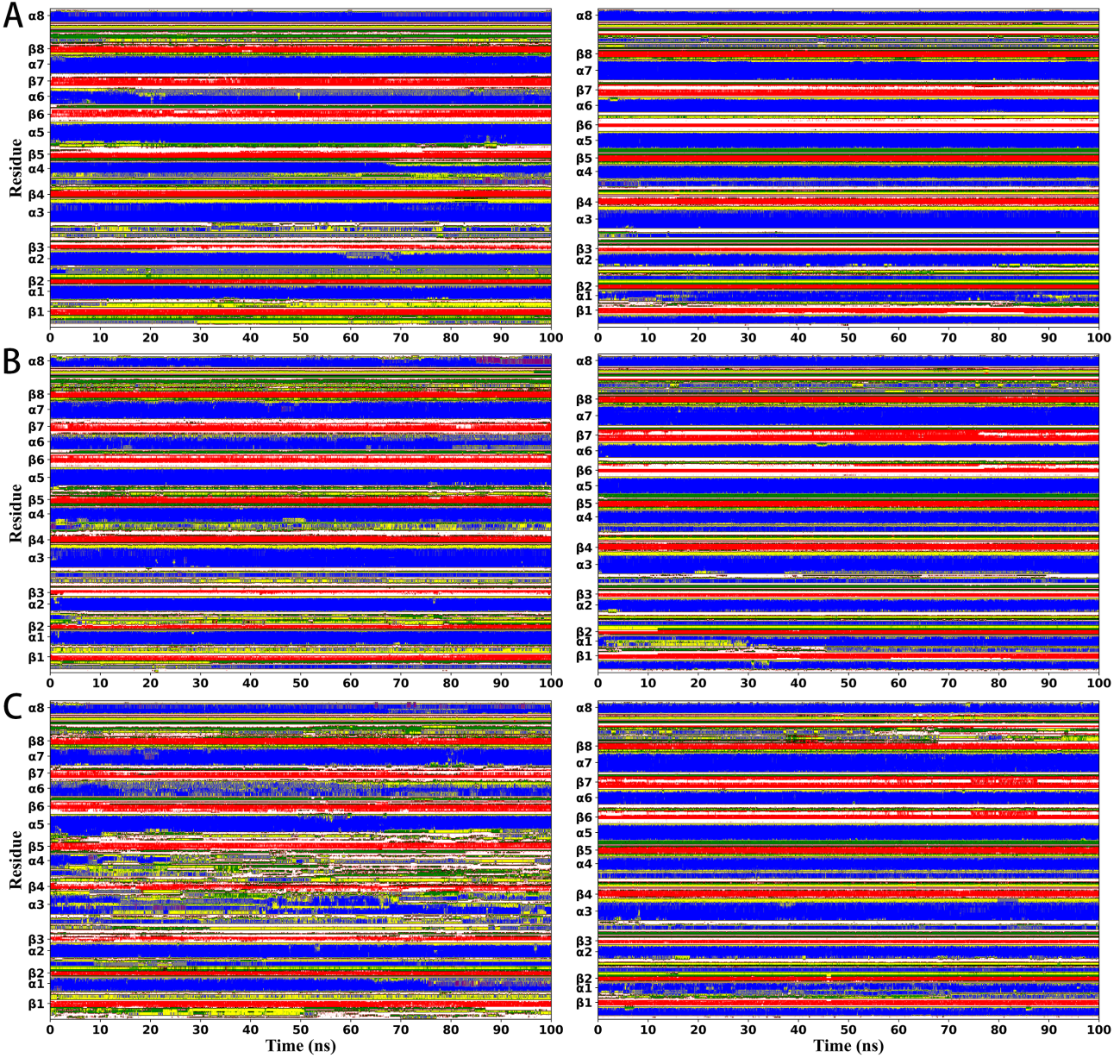


**SUPPLEMENTARY FIGURE S9**

Time evolution of secondary structure propensities of the XynDRTY1 (left) and XynM1 (right) from the third replica of the molecular dynamics simulation at 300 K (A), 350 K (B), and 400 K (C).
